# Comparative Study of Scientific Publications in Urology and Nephrology Journals Originating from USA, China and Japan (2001–2010)

**DOI:** 10.1371/journal.pone.0042200

**Published:** 2012-08-01

**Authors:** Juan Li, Xian Hua Gao, Qi Bian, Zhi Yong Guo, Xiao Bin Mei, Guang Yu, Hao Wu, Xue Li Lai, Wei Chen

**Affiliations:** 1 Department of Nephrology, Changhai Hospital, Second Military Medical University, Shanghai, China; 2 Department of Colorectal Surgery, Changhai Hospital, Second Military Medical University, Shanghai, China; National Cancer Institute, United States of America

## Abstract

**Background:**

In the past decade, scientific research has developed rapidly in China, but the growth seems to vary widely between different disciplines. In this study, we aimed to compare the quantity and quality of publications in urology and nephrology journals from USA, China and Japan.

**Methods:**

Journals listed in the “Urology and Nephrology” category of Science Citation Index Expanded subject categories were included. Scientific papers in these journals written by researchers from USA, Japan and China were retrieved from the “PubMed” and “Web of Knowledge” online databases.

**Results:**

The annual number of total scientific articles increased significantly from 2001 to 2010 in China, and has ranked second in the world since 2006. In the field of urology and nephrology, the annual number increased significantly from 2001 to 2010 in USA and China; but not in Japan. The share of articles increased significantly over time in China, decreased significantly in Japan, and remained unchanged in USA. In 2010, USA contributed 32.17% of the total world output in urology and nephrology field and ranked 1^st^; Japan contributed 5.19% and ranked 5^th^; China contributed 3.83% and ranked 9^th^. Publications from USA had the highest accumulated IFs and the highest total citations of articles (USA>Japan>China, p<0.001). No significant difference was found in average IF among the three countries. USA published the most articles in the top 10 urology and nephrology journals (USA(35165)>Japan(6704)>China(2233), p<0.001). Researchers from USA published more clinical trials and randomized controlled trials than Japan and China (USA>Japan>China, p<0.001).

**Conclusion:**

Although China has undergone significant increase in annual number and percentage of scientific publication in urology and nephrology journals in the past decade, it still lags far behind USA and Japan in the field of urology and nephrology in terms of quantity and quality.

## Introduction

The increasing incidence and prevalence of chronic kidney disease (CKD) has become a global public health challenge [1]. With more than 1.3 billion people, China is the world’s largest and most populous country. In China, the overall prevalence of chronic kidney disease is 10.8%, and the total number of patients with chronic kidney disease is estimated to be about 119.5 million [2], which is larger than that of USA (26.3 million) [3]. Renal replacement therapies (RRT), including maintenance peritoneal dialysis, hemodialysis and renal transplantation, are the required treatments for patients with End Stage Renal Disease (ESRD). About 102,700 patients received dialysis in China in 2008 [4], and about 5000 patients underwent renal transplantation every year in China [5]. As a large and growing clinical problem, RRT consumes a considerable proportion of health care resources, and has posed large economic burdens on patients’ families and the government [6]. In a word, chronic kidney disease has become an important public health problem in China [2].

The study of scientific publications in a particular field, based on international bibliographic data, is one of the widely used methods to measure scientific achievement [7]. The development of bibliographic computer retrieval system “Pubmed” database and citation based system “Web of knowledge” online database introduced by the Institute for Scientific Information [8], has improved the speed and precision of literature data collection and comparison. It is well documented that USA is the leading power in biomedical investigation and publications in most scientific disciplines [7]. Japan, as a neighboring country of China, is also among the top-ranking countries of scientific research [9]. In the past decade, we have witnessed the remarkable development of China in scientific research, which ranks second in annual total number of scientific publications in the world since 2006, second only to the USA [10]. So far, little is known about China’s scientific contribution in the field of urology and nephrology.

This study aimed to evaluate the quantity and quality of scientific publications in the field of urology and nephrology in China during the past decade (2001–2010), and to compare these with USA and Japan.

## Results

### Total Number of Scientific Articles in USA, China and Japan

**Figure 1:**
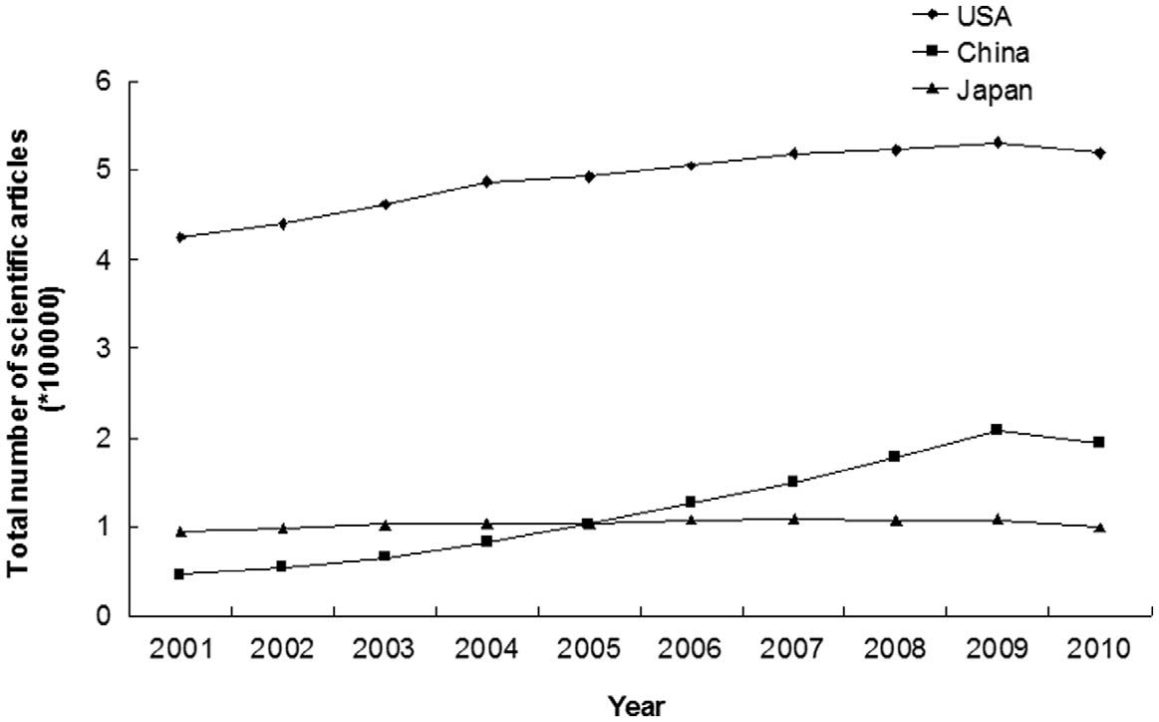
Trends in annual numbers of scientific articles published by researchers from USA, China and Japan (2001–2010).

**Figure 2:**
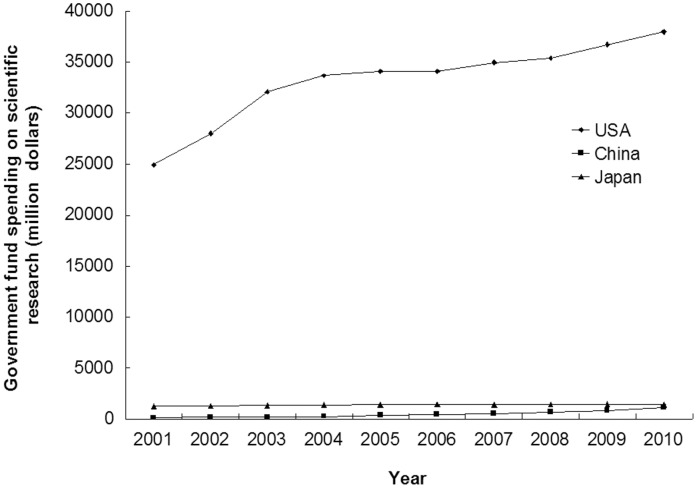
Government fund spending on scientific research from USA, China and Japan (2001–2010).

A total of 7148343 articles were published in the SCI-cited journals from 2001 to 2010 in the three countries; 29.69% of these were from USA (4902307/16512009), 7.33% were from China (1210113/16512009) and 6.27% were from Japan (1035923/16512009). The annual number of published scientific articles increased significantly from 2001 to 2010 in USA (424320 to 519741, annual incremental rate = 2.28%, r = 0.947, p<0.001) and China (46631 to 194551, annual incremental rate = 17.20%, r = 0.984, p<0.001); but not in Japan (94588 to 99634, annual incremental rate = 0.58%, r = 0.610, p = 0.061) (Fig. 1 and Table S1). The number of scientific articles from China has exceeded that from Japan since 2006, and ranked second in the world thereafter (Table S1). USA always ranked first in the production of scientific articles. The share of articles decreased significantly over time in USA (32.06% to 27.61%, annual incremental rate = −1.65%, r = 0.946, p<0.001) and Japan (7.15% to 5.29%, annual incremental rate = −3.29%, r = 0.979, p<0.001), but increased significantly over time in China (3.52% to 10.34%, annual incremental rate = 12.72%, r = 0.992, p<0.001) (Table S1). The government fund spending on scientific research increased slowly in USA (24939 to 37963 million dollars, annual incremental rate = 4.78%, r = 0.919, p<0.001) and Japan (1263 to 1401 million dollars, annual incremental rate = 1.16%, r = 0.856, p = 0.002), but increased rapidly in China (114 to 1125 million dollars, annual incremental rate = 28.97%, r = 0.971, p<0.001) (Figure 2, Table S2).

### Number of Articles in the Field of Urology and Nephrology in USA, China and Japan

**Figure 3:**
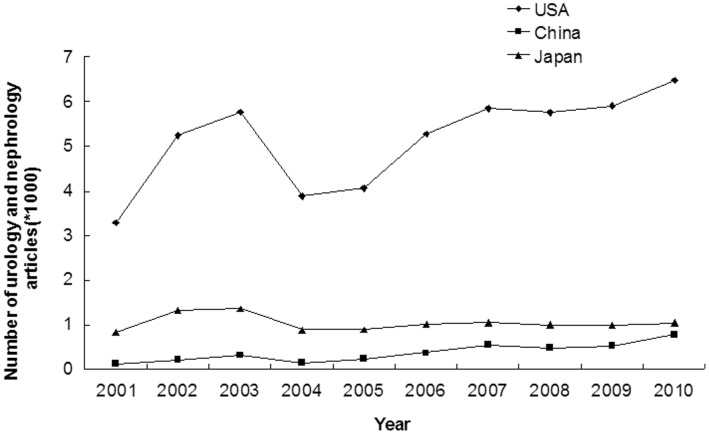
Annual numbers of articles in the 64 urology and nephrology journals written by researchers from USA, China and Japan (2001–2010).

**Figure 4:**
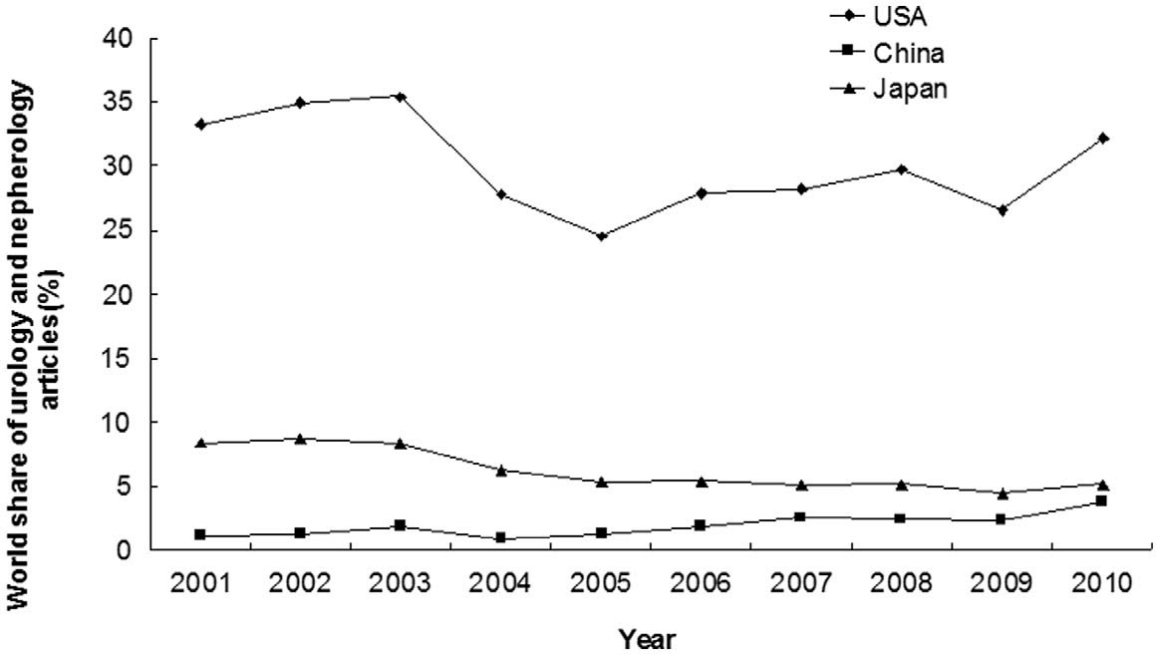
Annual proportion of articles in the 64 urology and nephrology journals written by researchers from USA, China and Japan (2001–2010).

A total of 65548 articles were published in the 64 journals (Table S3) by the three countries from 2001 to 2010; 78.56%(51497/65548) of these were from USA, 5.59%(3665/65548) were from China and 15.84%(10386/65548) were from Japan. The annual number of published articles in the field of urology and nephrology increased significantly from 2001 to 2010 in USA (3281 to 6481, annual incremental rate = 7.86%, r = 0.708, p = 0.022) and China (117 to 771, annual incremental rate = 23.31%, r = 0.903, p<0.001); but not in Japan (829 to 1045, annual incremental rate = 2.61%, r = 0.154, p = 0.671) (Fig. 3 and Table S3). The share of articles increased significantly over time in China (1.19% to 3.83%, annual incremental rate = 13.87%, r = 0.838, p = 0.002), and decreased significantly in Japan (8.42% to 5.19%, annual incremental rate = −5.23%, r = 0.887, p = 0.001), and remained unchanged in USA (33.31% to 32.17%, annual incremental rate = −0.39%, r = 0.459, p = 0.182) (Fig. 4 and Table S3). In 2010, USA contributed 32.17% of the total world output in urology and nephrology field and ranked 1^st^; Japan contributed 5.19% and ranked 5^th^; China contributed 3.83% and ranked 9^th^ (Table S3, S4).

### Clinical Trials, Randomized Controlled Trials (RCT) and Case Reports (Fig. 5)

**Figure 5:**
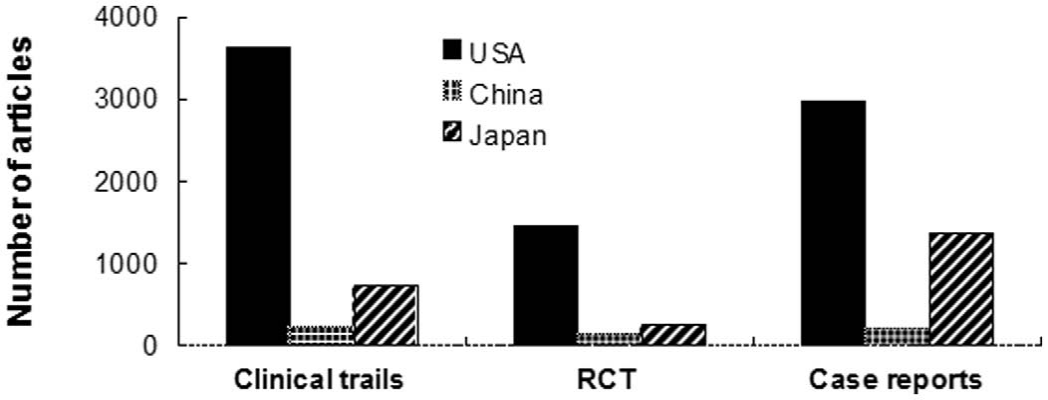
Number of clinical trials, randomized controlled trials (RCT), and case reports published by researchers from USA, China, and Japan from 2001 to 2010.

Researchers from USA published more clinical trials than those from Japan and China (USA(3630)>Japan(717)>China(219), all p values were less than 0.001, Fig. 5), surpassing the combined number of Japan and China. Researchers from USA published 1460 RCTs between 2001 and 2010, which far exceeded those from China (n = 123, p<0.001) and Japan (n = 233, p<0.001). The number of RCTs from Japan was significantly larger than this from China (p<0.001). The numbers of case reports from USA, China and Japan differed significantly (USA(2983)>Japan(1366)>China(186), all p values were less than 0.001 ) (Fig. 5).

### Impact Factors

According to the JCR, the 64 urology and nephrology journals had IFs in 2010 [10]. The accumulated IF of articles from USA were much higher than that of Japan and China (204443.6 vs. 33794.8 vs. 11295.8, all p values were less than 0.001). However, the average IF of urology and nephrology articles from USA, China and Japan did not differ significantly (p>0.05, Table 1).

**Table 1:** The accumulated and average impact factors of articles published in urology and nephrology journals by researchers from USA, China, and Japan from 2001 to 2010.

	Accumulated impact factor			Average impact factor		
Year	USA	China	Japan	USA	China	Japan
2001	11926.3	301.2	2375.5	3.79	2.95	3.30
2002	28221.4	1187.0	6054.1	5.54	6.21	5.16
2003	31510.4	1664.0	6765.1	5.53	5.57	5.09
2004	14337.6	458.7	2706.5	3.71	3.30	3.07
2005	15285.9	719.9	2710.2	3.76	3.32	3.11
2006	18639.2	1086.8	2808.5	3.61	3.00	2.77
2007	20514.7	1358.3	2821.8	3.51	2.51	2.68
2008	20733.7	1236.7	2523.1	3.60	2.59	2.52
2009	21256.3	1396.4	2483.8	3.65	2.69	2.57
2010	22018.1	1886.9	2546.2	3.43	2.49	2.47
Total	204443.6	11295.8	33794.8	40.12	34.63	32.74

### Citations of Articles Published in Urology and Nephrology Journals

**Figure 6:**
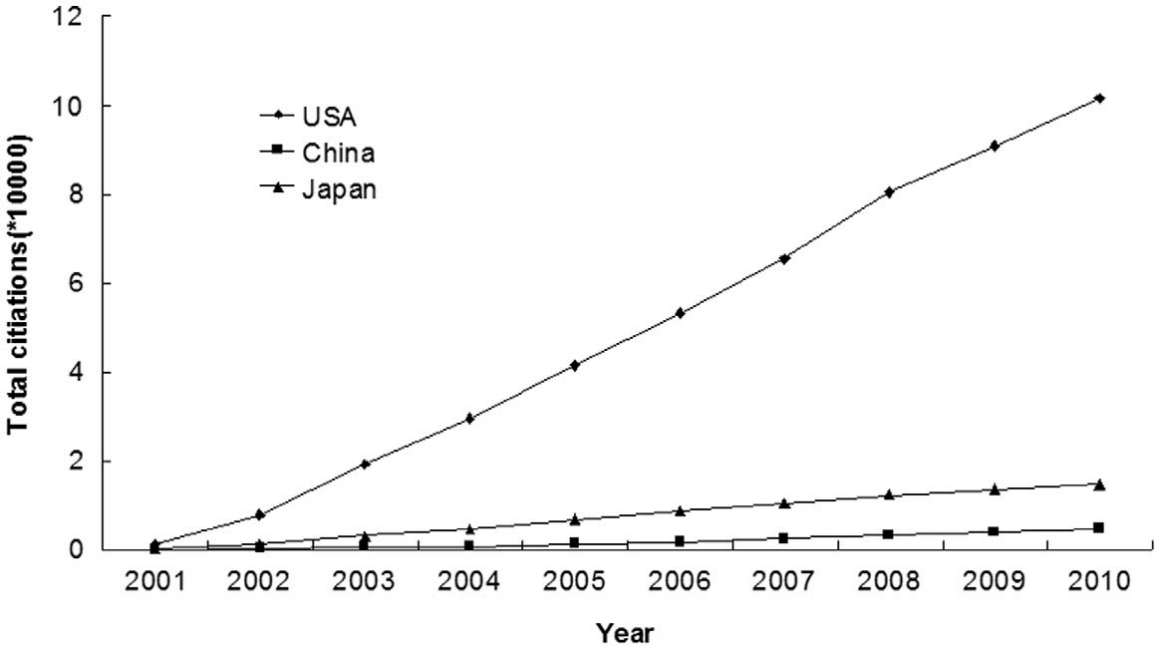
Annual citations of articles published in urology and nephrology journals by researchers from USA, China, and Japan from 2001 to 2010.

Articles from USA were most cited (1016135 citations), followed by those from Japan (14732 citations) and China (4762 citations). These differences among the three countries were all significant (p<0.001, Fig. 6).

### Articles in the 10 Top-ranking Urology and Nephrology Journals

A total of 44102 articles from the three countries were published in the 10 top-ranking urology and nephrology journals. Among them, 36.7% (16180/44102) were in the top three journals: *European Urology, Journal of the American Society of Nephrology, and Kidney International*. Researchers from USA published 35165 (79.74%) articles in 10 high-impact urology and nephrology journals, those from Japan published 2233 (5.06%) articles, and those from China published 6704 (15.2%) articles (Table 2).

**Table 2:** Articles published in the 10 highest-impact nephrology and urology journals by researchers from USA, China and Japan from 2001 to 2010.

Rank	Journal	2010IF	USA(%)	China(%)	Japan(%)	Total
1	EUR UROL	8.84	6245(79.49)	348(4.43)	1263(16.08)	7856
2	J AM SOC NEPHROL	8.29	3046(79.28)	202(5.26)	594(15.46)	3842
3	KIDNEY INT	6.11	3657(81.59)	221(4.93)	604(13.48)	4482
4	NAT CLIN PRACT NEPHR	5.53	1349(72.57)	162(8.71)	348(18.72)	1859
5	AM J KIDNEY DIS	5.24	8160(80.98)	358(3.55)	1558(15.46)	10076
6	CLIN J AM SOC NEPHRO	4.76	2278(79.62)	176(6.15)	407(14.23)	2861
7	NAT REV NEPHROL	4.75	1290(74.74)	133(7.71)	303(17.56)	1726
8	CURR OPIN NEPHROL HY	4.46	1847(76.32)	173(7.15)	400(16.53)	2420
9	J SEX MED	3.96	4277(80.64)	277(5.22)	750(14.14)	5304
10	J UROLOGY	3.86	3016(82.05)	183(4.98)	477(12.98)	3676
Total			35165(79.74)	2233(5.06)	6704(15.2)	44102

### Popular Urology and Nephrology Journals

The journals that published the most articles written by researchers from the three countries are listed in Table 3. Most articles from USA were published in *Journal of Urology*, most articles from China were published in *Journal of the American Society of Nephrology*, and most articles from Japan were published in *International Journal of Urology*. *Journal of Urology*, *Urology* and *Kidney International* appeared among the 10 top popular journals for all three countries.

**Table 3:** The 10 nephrology and urology journals publishing the most articles written by researchers from USA, China and Japan.

Rank	USA(IF)	N	China(IF)	N	Japan(IF)	N
1	JU(3.86)	8160	JASN(8.29)	358	IJU(1.46)	1558
2	JASN(8.29)	6245	JE(1.73)	348	JSM(3.96)	1263
3	Urology(2.33)	4277	AJA(1.55)	277	N(1.17)	750
4	AJKD(5.24)	3657	NDT(3.56)	221	KI(6.11)	604
5	KI(6.11)	3046	PN(2.18)	202	JU(3.86)	594
6	JE(1.73)	3016	N(1.17)	183	Urology (2.33)	477
7	AJPRP(3.79)	2278	KI(6.11)	176	PN(2.18)	407
8	BJUI(3.19)	1847	PDI(1.48)	173	TAD(1.1)	400
9	JSM(3.96)	1349	Urology(2.33)	162	CN(1.06)	348
10	NDT(3.56)	1290	JU(3.86)	133	AJKD(5.24)	303
Total		35165		2233		6704

JU: Journal of Urology, JASN: Journal of the American Society of Nephrology, AJKD: American Journal of Kidney Diseases, KI: Kidney International, JE: Journal of Endourology, AJPRP: American Journal of Physiology Renal Physiology, BJUI: Bju International, JSM: Journal of Sexual Medicine, NDT: Nephrology Dialysis Transplantation, AJA: Asian Journal of Andrology, PN: Pediatric Nephrology, N: Nephrology, PDI: Peritoneal Dialysis International, IJU: International Journal of Urology, TAD: Therapeutic Apheresis and Dialysis, CN: Clinical Nephrology.

## Discussion

USA, China and Japan are all major countries in the world in terms of population, economy and scientific research. As two of the most developed countries, USA and Japan have been in the forefront of global scientific research for many years. China has changed a great deal in the past decade, with rapid development in education, urbanization and economy, and scientific research has improved greatly. China has been ranking second in annual total number of scientific papers published in SCI-cited journals since 2006. However, this growth seemed to vary greatly between different disciplines in China. To the best of our knowledge, this is the first report to compare scientific publications from the three countries in the urology and nephrology field.

Our study showed the total number and the percentage of scientific publications from USA were the highest in the world, and the results were similar in the urology and nephrology field. From 2001 to 2010, the number of articles of USA in the urology and nephrology field had increased significantly, and its percentage of research articles remained unchanged. The number of articles published in scientific journal is a reflection of research activity in a country [11], [12]. Therefore, there is no doubt that USA leads all other countries in scientific publication productivity in the urology and nephrology field [13], [14]. The active role of USA in the scientific research may be attributed to the following factors: long working hours, strong individual competition for tenure, strict and objective peer review, bottom-up initiatives, generous economic support, several interactive research environments, and frequent exchange of foreign students and young investigators [15]. Although the annual number of published articles in the field of urology and nephrology increased slightly in Japan without statistic significance, the share of articles declined from 8.42% in 2001 to 5.19% in 2010 (p = 0.001). However, there is no doubt that the Japan still plays an important role in the urology and nephrology field. The annual total number of scientific publications in China increased rapidly in the past decade, had surpassed those of Japan since 2006, and ranked second in the world thereafter. With great improvements in policy reform, meteoric economic rise, modernization of diagnostic and therapeutic methods, increase in the number of researchers and research fund, and more frequent international collaboration [16], marked development in scientific publications has also inevitably taken place [17]. From 2001 to 2010, the government fund spending on scientific research increased rapidly in China (114 million dollars in 2001 to 1125 million dollars in 2010), with an annual incremental rate of 28.97% (Figure 2, Table S2). Comparing the Figure 1 and Figure 2, we could find that the changing trends of scientific publication outputs were similar to the changing trends of government scientific funds. Therefore, our results indicated scientific publication outputs were closely related with the amount of government research fund. The rapid increase of scientific publications in China may be attributed to the high increasing rate (annual incremental rate = 28.97%) of scientific funds.

An interesting finding of this study is the total number and percentage of articles in urology and nephrology journals from China increased significantly during the last decade. Our study demonstrated that the absolute number of Chinese articles in urology and nephrology journals had a 7-fold increase (from 117 papers in 2001 to 771 paper in 2010, p<0.001), and the share of articles also increased significantly in China (from 1.19% in 2001 to 3.83% in 2010, p = 0.002). China’s scientific research has been growing rapidly in the past decade, but the growth seems to vary widely between different disciplines [18]. In urology and nephrology field, even in 2010 China only contributed 3.83% of the total world output and ranked 9^th^, lagging far behind USA (32.17%) and Japan (5.19%). That is to say, China remains one of the smaller players in the Urology and Nephrology field, with its share of total publications of 3.83%, which is less than the 10% in materials science and the 8% in mathematics and physics [18]. There are many causes attributing to the low quantity of scientific publications in Urology and Nephrology field in China. Firstly, the relatively low amount of government scientific fund in medicine is a major reason. In the USA, government funds on medicine are operating by an independent institution, the National Institution of Health; while government funds on other disciplines are operated by US National Scientific Foundation. Government funds on medical research account for more than 80% of the total funds, which are greatly larger than the total funds on other disciplines. In China, government funds on medical research only account for 20–30% of the total government funds. Secondly, the relative late initiation of this discipline is also an important reason. Nephrological work in China started in early 60s, but it was not until the middle of the 1980s before it became an independent discipline and linked with the international nephrology community [5]. Thirdly, the great disparity between urban and vast rural areas in China is also an important cause. Although China has gained great achievement in economy in the past decade, most population in the rural areas are still in poverty. More than 50% of the rural population can not afford any kind of medical care [19], [20]. So, nephrology development is at a relatively low level in the rural areas, far from the modernization level to publish scientific articles in international journals. Fourthly, the use of English as the language of publication for most scientific publications is also an important disturbance for Chinese researchers. And a substantial number of articles by Chinese authors are published in journals in Chinese. Fifthly, the lack of genetic mouse model in china as compared to Japan and USA may also be an important factor for Chinese retardation in basic science research.

Randomized Controlled Trials (RCT) and clinical trials, which are associated with a higher grade of evidence, are more helpful for the improvement of diagnosis and treatment. Our study documented that China lagged far behind USA and Japan in the number of RCTs and clinical trials (USA>Japan>China, p<0.001). The prevalence and burden of chronic disease will likely continue to grow in the future [4]. Thus, Chinese researchers should prioritize more RCTs and clinical trails which are more helpful to promote the development of nephrology.

The impact factor (IF) indicates the average number of citations to articles in publications. Although IF is not an appropriate measure of the scientific quality of individual articles [21], it is still one of the most useful tools to gauge the relative importance of scientific researches [22]. Our study showed that there was no significant difference among the three countries in average impact factors (p>0.05), suggesting research quality is comparable among China, Japan and USA. The phenomena may have something to do with the fact that most nephrologists of China reside in metropolis of the coastal region, which has a higher education background than most of the rest of the country [5]. It is reported that the research output of China was mainly from five cities, Beijing, Shanghai, Nanjing, Guangzhou and Hangzhou, which accounted for 72% of the total amount [23]. However, the accumulated impact factor of USA is much larger than those of China and Japan (p<0.001). Another important indicator for article quality is analysis of citation indices. The number of citations an article receives reflects its scientific impact to some extent [15]. Our study demonstrated that articles from USA were cited most, those from Japan were cited second-most, and those from China were cited the least; all of the differences were statistically significant (p<0.001). As far as the top 10 urology and nephrology journals were concerned, researchers from USA and Japan also published more papers than those from China (p<0.001). In summary, our comparison of publication quality using IF, citation index, number of clinical trails, number of RCTs and number of articles published in the top 10 journals demonstrated that China still lagged far behind USA and Japan even at the end of the study period (2010).

There are some inherent limitations in this study. Firstly, the urology and nephrology journals were selected from the “urology and nephrology” category of Science Citation Index Expanded (SCIE) for 2010. The included journals have been changing year by year, although most of journals remained unchanged. In addition, some relevant journals were not included in the urology and nephrology category of the SCIE. Secondly, we limited the author’s affiliation to country names (USA, China, Japan), which would omit articles that did not designate country names. For some studies that were conducted in joint collaboration with other regions or countries, only corresponding authors affiliations were included as the origin of research in the PubMed database, which neglected the contributions of other researchers from different geographic areas. Therefore, the absolute numbers of articles in the urology and nephrology field originating from the three countries are certainly different from our findings. Thirdly, “the accumulated impact factors (IF) and the average IF” were evaluated by utilizing the IFs of JCR 2010. In the past decade, the IFs of the journals had changed year by year. Therefore, the accumulated IF and average IF reported in this study is only estimation, but it is likely to reflect the trend since the alteration of IF is relatively slight for most journals in the past decade. However, despite these limitations, we believe that the results in this study are likely to reflect the real situation of urology and nephrology research in USA, China and Japan.

In conclusion, our study demonstrated that the annual total number of scientific articles from China has increased significantly in the past decade, and exceeded Japan and ranked second in the world since 2006. In the field of urology and nephrology, China has made a remarkable progress in annual number and percentage of scientific publication in the past decade (2001–2010). The results of this study also imply that China still lags far behind USA and Japan in this field. The quantity and quality of urology and nephrology articles need to be improved. Remedial measures should be taken for China to promote scientific researches in the urology and nephrology field. It is also important for China to galvanize research with higher grade evidence such as clinical trails and RCTs.

## Materials and Methods

This retrospective study examined 69 journals related to urology and nephrology that were selected from the “urology and nephrology systems” category of the Science Citation Index Expanded (SCIE) for 2010 [24]. This category included resources for the diagnosis and treatment of genitourinary and kidney diseases: general urology and nephrology publications, and specialized research on the kidney diseases, renal failure, peritoneal dialysis, hemodialysis and renal transplantation, and disease of the ureter, bladder and prostate. *European Urology Supplements*, *Néphrologie & Thérapeutique*, *pelvi-perineologie*, *Revista de Nefrologia Dialisis y Trasplante* and *Annales D Urologie* were not indexed by Medline and were excluded from this study. Our search of the “Pubmed” and “Web of knowledge” database on December 18^th^, 2011 sought articles published in the 64 journals (Table S5) between January 1^st^ 2001 and December 31^th^ 2010 by researchers from USA, China and Japan [25]. The ISSN numbers of the journals (Table S5) were used to perform this search. Scientific output from the three countries was identified using the authors’ institutional affiliations. Original clinical trials, randomized controlled trials (RCT) and case reports were compiled using the publication type categories of the PubMed database. We used online database (US National Science Foundation [26], National Institution of Health [27], Nature Science Foundation of China [28], Japan Science and Technology Agency (JST) [29]), to retrieve information on government fund spending on scientific research, and percentage of medical research fund.

Five methods were used to compare publication quality. Firstly, the accumulated and average IFs were determined using the ISI’s 2010 Journal Citation Reports (JCR) [10]. Secondly, we quantified citations of articles written by researchers from the three countries. Thirdly, we calculated the number of Randomized Controlled Trials (RCT) and clinical trials, which were associated with a higher grade of evidence. Fourthly, the number of articles published by each country in the top 10 high-impact urology and nephrology journals was also compared. Fifthly, we determined the top 10 popular urology and nephrology journals for the three countries according to the number of articles published by each journal.

### Statistical Analyses

Statistical analyses were performed using SPSS 17.0 (SPSS, Chicago, IL). Count data were summarized in the Tables and Figures. Annual incremental rate was used to reflect the changing trend of scientific publications and government research fund from 2001 to 2010. The linear regression analysis was performed to determine any significant change of the total numbers over the period of time. “r” means correlation coefficient. The Kruskal-Wallis test was used to detect differences among the three countries, and rank-sum tests were conducted for detecting the differences between two countries when necessary. The test for significance was two-tailed and p<0.05 was considered significant.

## Supporting Information

Table S1 Numbers of scientific articles written by researchers from USA, China and Japan from 2001 to 2010.
(DOC)

Table S2 Government fund (million dollars) spending on scientific research from USA, China and Japan (2001–2010).
(DOC)

Table S3 Numbers of articles in urology and nephrology journals written by researchers from USA, China and Japan from 2001 to 2010.
(DOC)

Table S4 The top 10 countries in output of scientific articles in urology and nephrology journals from 2001 to 2010.
(DOC)

Table S5 The journal title, impact factor and ISSN of the included 64 journals.
(DOC)
