# Supplementary material for: Comparative Study of Scientific Publications in Urology and Nephrology Journals Originating from USA, China and Japan (2001–2010)
Source: PLoS One. 2012 Aug 1;7(8):e42200. doi: 10.1371/journal.pone.0042200 (PMC3411650; doi:10.1371/journal.pone.0042200)
Supplement: Table S1 — Numbers of scientific articles written by researchers from USA, China and Japan from 2001 to 2010. (DOC) [file pone.0042200.s001.doc]

| Year | USA | | | China | | | Japan | | | The Whole World |
| --- | --- | --- | --- | --- | --- | --- | --- | --- | --- | --- |
|  | Number | Percentage | Rank | Number | Percentage | Rank | Number | Percentage | Rank |  |
| 2001 | 424320 | 32.06% | 1 | 46631 | 3.52% | 7 | 94588 | 7.15% | 3 | 1,323,386 |
| 2002 | 440306 | 32.12% | 1 | 53517 | 3.90% | 6 | 97673 | 7.13% | 2 | 1,370,880 |
| 2003 | 460864 | 32.32% | 1 | 65800 | 4.62% | 5 | 102669 | 7.20% | 2 | 1,425,853 |
| 2004 | 486959 | 32.10% | 1 | 83087 | 5.48% | 5 | 103628 | 6.83% | 2 | 1,517,197 |
| 2005 | 492711 | 30.56% | 1 | 103202 | 6.40% | 5 | 103367 | 6.41% | 4 | 1,612,345 |
| 2006 | 504705 | 29.85% | 1 | 127296 | 7.53% | 2 | 108200 | 6.40% | 5 | 1,691,049 |
| 2007 | 518896 | 28.48% | 1 | 150333 | 8.25% | 2 | 110016 | 6.04% | 5 | 1,821,808 |
| 2008 | 522823 | 27.53% | 1 | 177614 | 9.35% | 2 | 107581 | 5.67% | 5 | 1,898,888 |
| 2009 | 530982 | 26.98% | 1 | 208082 | 10.57% | 2 | 108567 | 5.52% | 5 | 1,968,413 |
| 2010 | 519741 | 27.61% | 1 | 194551 | 10.34% | 2 | 99634 | 5.29% | 5 | 1,882,190 |
| Total | 4902307 | 29.69% |  | 1210113 | 7.33% |  | 1035923 | 6.27% |  | 16,512,009 |
