# Supplementary material for: Comparative Study of Scientific Publications in Urology and Nephrology Journals Originating from USA, China and Japan (2001–2010)
Source: PLoS One. 2012 Aug 1;7(8):e42200. doi: 10.1371/journal.pone.0042200 (PMC3411650; doi:10.1371/journal.pone.0042200)
Supplement: Table S2 — Government fund (million dollars) spending on scientific research from USA, China and Japan (2001–2010). (DOC) [file pone.0042200.s002.doc]

| Year | USA | | | China | | | Japan |
| --- | --- | --- | --- | --- | --- | --- | --- |
|  | Total^a^ | Medical^b^ | Percentage^c^ | Total | Medical | Percentage | Total |
| 2001 | 24939 | 20513 | 82.25% | 114 | 33 | 29.30% | 1263 |
| 2002 | 27978 | 23188 | 82.88% | 165 | 47 | 28.52% | 1302 |
| 2003 | 32085 | 26740 | 83.34% | 189 | 54 | 28.67% | 1340 |
| 2004 | 33678 | 28100 | 83.44% | 239 | 74 | 30.99% | 1379 |
| 2005 | 34099 | 28626 | 83.95% | 419 | 104 | 24.71% | 1417 |
| 2006 | 34114 | 28533 | 83.64% | 496 | 142 | 28.72% | 1429 |
| 2007 | 34951 | 29034 | 83.07% | 571 | 165 | 28.95% | 1423 |
| 2008 | 35385 | 29320 | 82.86% | 735 | 215 | 29.29% | 1438 |
| 2009 | 36697 | 30207 | 82.31% | 831 | 246 | 29.63% | 1466 |
| 2010 | 37963 | 31036 | 81.75% | 1125 | 231 | 20.57% | 1401 |
| Total | 331888 | 275297 | 82.95% | 4884 | 1313 | 26.88% | 13858 |
| ^a^ “Total” means total government fund spending on scientific research;  ^b^ “Medical” means government fund spending on medical research;  ^c^ “Percentage” means the share of medical government fund in total government fund. | | | | | | | |
