# Supplementary material for: Comparative Study of Scientific Publications in Urology and Nephrology Journals Originating from USA, China and Japan (2001–2010)
Source: PLoS One. 2012 Aug 1;7(8):e42200. doi: 10.1371/journal.pone.0042200 (PMC3411650; doi:10.1371/journal.pone.0042200)
Supplement: Table S3 — Numbers of articles in urology and nephrology journals written by researchers from USA, China and Japan from 2001 to 2010. (DOC) [file pone.0042200.s003.doc]

| Year | Articles of USA | | | Articles of China | | | Articles of Japan | | | Total Number* |
| --- | --- | --- | --- | --- | --- | --- | --- | --- | --- | --- |
|  | Number | Percentage | Rank | Number | Percentage | Rank | Number | Percentage | Rank |  |
| 2001 | 3,281 | 33.31% | 1 | 117 | 1.19% | 18 | 829 | 8.42% | 3 | 9,850 |
| 2002 | 5,249 | 34.92% | 1 | 200 | 1.33% | 15 | 1320 | 8.78% | 2 | 15,030 |
| 2003 | 5,765 | 35.44% | 1 | 310 | 1.91% | 10 | 1360 | 8.36% | 2 | 16,267 |
| 2004 | 3,877 | 27.87% | 1 | 139 | 1.00% | 19 | 878 | 6.31% | 3 | 13,910 |
| 2005 | 4,067 | 24.53% | 1 | 217 | 1.31% | 15 | 888 | 5.36% | 4 | 16,578 |
| 2006 | 5,272 | 27.96% | 1 | 362 | 1.92% | 11 | 1018 | 5.40% | 4 | 18,858 |
| 2007 | 5,849 | 28.27% | 1 | 543 | 2.62% | 11 | 1052 | 5.08% | 5 | 20,693 |
| 2008 | 5,757 | 29.75% | 1 | 478 | 2.47% | 11 | 1002 | 5.18% | 5 | 19,352 |
| 2009 | 5,899 | 26.59% | 1 | 528 | 2.38% | 12 | 994 | 4.48% | 5 | 22,189 |
| 2010 | 6,481 | 32.17% | 1 | 771 | 3.83% | 9 | 1045 | 5.19% | 5 | 20,145 |
| Total | 51497 | 29.79% |  | 3665 | 2.12% |  | 10386 | 6.01% |  | 172872 |
| * The total number of articles published in the 64 Urology and Nephrology journals from 2001 to 2010. | | | | | | | | | | |
