# Supplementary material for: Comparative Study of Scientific Publications in Urology and Nephrology Journals Originating from USA, China and Japan (2001–2010)
Source: PLoS One. 2012 Aug 1;7(8):e42200. doi: 10.1371/journal.pone.0042200 (PMC3411650; doi:10.1371/journal.pone.0042200)
Supplement: Table S4 — The top 10 countries in output of scientific articles in urology and nephrology journals from 2001 to 2010. (DOC) [file pone.0042200.s004.doc]

| Year | Rank | Country | Number | Percentage | Year | Rank | Country | Number | Percentage |
| --- | --- | --- | --- | --- | --- | --- | --- | --- | --- |
| 2001 | 1 | **USA** | 3281 | 33.31% | 2006 | 1 | **USA** | 5272 | 27.96% |
|  | 2 | GERMANY | 929 | 9.43% |  | 2 | GERMANY | 1210 | 6.42% |
|  | 3 | **JAPAN** | 828 | 8.41% |  | 3 | ENGLAND | 1103 | 5.85% |
|  | 4 | FRANCE | 613 | 6.22% |  | 4 | **JAPAN** | 1017 | 5.39% |
|  | 5 | ENGLAND | 606 | 6.15% |  | 5 | ITALY | 944 | 5.01% |
|  | 6 | ITALY | 554 | 5.62% |  | 6 | FRANCE | 654 | 3.47% |
|  | 7 | SPAIN | 327 | 3.32% |  | 7 | TURKEY | 639 | 3.39% |
|  | 8 | NETHERLANDS | 299 | 3.04% |  | 8 | CANADA | 602 | 3.19% |
|  | 9 | CANADA | 291 | 2.95% |  | 9 | SPAIN | 503 | 2.67% |
|  | 10 | SWEDEN | 216 | 2.19% |  | 10 | NETHERLANDS | 420 | 2.23% |
| 2002 | 1 | **USA** | 5249 | 34.92% | 2007 | 1 | **USA** | 5849 | 28.27% |
|  | 2 | **JAPAN** | 1316 | 8.76% |  | 2 | GERMANY | 1698 | 8.21% |
|  | 3 | GERMANY | 1100 | 7.32% |  | 3 | ENGLAND | 1478 | 7.14% |
|  | 4 | ENGLAND | 745 | 4.96% |  | 4 | ITALY | 1154 | 5.58% |
|  | 5 | ITALY | 684 | 4.55% |  | 5 | **JAPAN** | 1051 | 5.08% |
|  | 6 | FRANCE | 678 | 4.51% |  | 6 | FRANCE | 980 | 4.74% |
|  | 7 | CANADA | 490 | 3.26% |  | 7 | CANADA | 741 | 3.58% |
|  | 8 | SPAIN | 434 | 2.89% |  | 8 | TURKEY | 680 | 3.29% |
|  | 9 | NETHERLANDS | 399 | 2.66% |  | 9 | SPAIN | 617 | 2.98% |
|  | 10 | AUSTRALIA | 277 | 1.84% |  | 10 | NETHERLANDS | 564 | 2.73% |
| 2003 | 1 | **USA** | 5765 | 35.44% | 2008 | 1 | **USA** | 5757 | 29.75% |
|  | 2 | **JAPAN** | 1355 | 8.33% |  | 2 | GERMANY | 1379 | 7.13% |
|  | 3 | GERMANY | 1206 | 7.41% |  | 3 | ENGLAND | 1242 | 6.42% |
|  | 4 | ENGLAND | 934 | 5.74% |  | 4 | ITALY | 1063 | 5.49% |
|  | 5 | FRANCE | 669 | 4.11% |  | 5 | **JAPAN** | 999 | 5.16% |
|  | 6 | ITALY | 655 | 4.03% |  | 6 | FRANCE | 937 | 4.84% |
|  | 7 | CANADA | 558 | 3.43% |  | 7 | CANADA | 824 | 4.26% |
|  | 8 | NETHERLANDS | 461 | 2.83% |  | 8 | SPAIN | 727 | 3.76% |
|  | 9 | SPAIN | 389 | 2.39% |  | 9 | TURKEY | 567 | 2.93% |
|  | 10 | **CHINA** | 295 | 1.81% |  | 10 | NETHERLANDS | 534 | 2.76% |
| 2004 | 1 | **USA** | 3877 | 27.87% | 2009 | 1 | **USA** | 5899 | 26.59% |
|  | 2 | GERMANY | 985 | 7.08% |  | 2 | GERMANY | 1474 | 6.64% |
|  | 3 | **JAPAN** | 878 | 6.31% |  | 3 | ENGLAND | 1299 | 5.85% |
|  | 4 | ENGLAND | 843 | 6.06% |  | 4 | ITALY | 1174 | 5.29% |
|  | 5 | ITALY | 636 | 4.57% |  | 5 | **JAPAN** | 993 | 4.48% |
|  | 6 | FRANCE | 574 | 4.13% |  | 6 | FRANCE | 989 | 4.46% |
|  | 7 | CANADA | 386 | 2.78% |  | 7 | CANADA | 877 | 3.95% |
|  | 8 | SPAIN | 355 | 2.55% |  | 8 | SPAIN | 672 | 3.03% |
|  | 9 | NETHERLANDS | 348 | 2.50% |  | 9 | NETHERLANDS | 671 | 3.02% |
|  | 10 | TURKEY | 343 | 2.47% |  | 10 | AUSTRALIA | 612 | 2.76% |
| 2005 | 1 | **USA** | 4067 | 24.53% | 2010 | 1 | **USA** | 6481 | 32.17% |
|  | 2 | ENGLAND | 1024 | 6.18% |  | 2 | GERMANY | 1308 | 6.49% |
|  | 3 | GERMANY | 1019 | 6.15% |  | 3 | ENGLAND | 1260 | 6.26% |
|  | 4 | **JAPAN** | 887 | 5.35% |  | 4 | ITALY | 1077 | 5.35% |
|  | 5 | ITALY | 760 | 4.58% |  | 5 | **JAPAN** | 1045 | 5.19% |
|  | 6 | FRANCE | 593 | 3.58% |  | 6 | CANADA | 992 | 4.92% |
|  | 7 | TURKEY | 487 | 2.94% |  | 7 | FRANCE | 919 | 4.56% |
|  | 8 | SPAIN | 442 | 2.67% |  | 8 | TURKEY | 739 | 3.67% |
|  | 9 | CANADA | 400 | 2.41% |  | 9 | **CHINA** | 725 | 3.60% |
|  | 10 | NETHERLANDS | 378 | 2.28% |  | 10 | SPAIN | 715 | 3.55% |
