# Supplementary material for: Comparative Study of Scientific Publications in Urology and Nephrology Journals Originating from USA, China and Japan (2001–2010)
Source: PLoS One. 2012 Aug 1;7(8):e42200. doi: 10.1371/journal.pone.0042200 (PMC3411650; doi:10.1371/journal.pone.0042200)
Supplement: Table S5 — The journal title, impact factor and ISSN of the included 64 journals. (DOC) [file pone.0042200.s005.doc]

| Rank | Full Journal Title | Impact Factor | ISSN |
| --- | --- | --- | --- |
| 1 | European Urology | 8.843 | 0302-2838 |
| 2 | Journal of the American Society of Nephrology | 8.288 | 1046-6673 |
| 3 | Kidney International | 6.105 | 0085-2538 |
| 4 | Nature Clinical Practice Nephrology | 5.525 | 1745-8323 |
| 5 | American Journal of Kidney Diseases | 5.242 | 0272-6386 |
| 6 | Clinical Journal of the American Society of Nephrology | 4.763 | 1555-9041 |
| 7 | Nature Reviews Nephrology | 4.750 | 1759-5061 |
| 8 | Current Opinion in Nephrology and Hypertension | 4.457 | 1062-4821 |
| 9 | Journal of Sexual Medicine | 3.957 | 1743-6095 |
| 10 | Journal of Urology | 3.862 | 0022-5347 |
| 11 | American Journal of Physiology Renal Physiology | 3.792 | 1931-857X |
| 12 | Nephrology Dialysis Transplantation | 3.564 | 0931-0509 |
| 13 | Prostate | 3.377 | 0270-4137 |
| 14 | BJU International | 3.190 | 1464-4096 |
| 15 | Urologic Oncology Seminars and Original Investigations | 3.172 | 1078-1439 |
| 16 | Nature Clinical Practice Urology | 3.130 | 1743-4270 |
| 17 | Neurourology and Urodynamics | 2.903 | 0733-2467 |
| 18 | Nature Reviews Urology | 2.891 | 1759-4812 |
| 19 | Current Opinion in Urology | 2.777 | 0963-0643 |
| 20 | Nephron Experimental Nephrology | 2.743 | 1660-2129 |
| 21 | American Journal of Nephrology | 2.658 | 0250-8095 |
| 22 | Nephron Physiology | 2.465 | 1660-2137 |
| 23 | World Journal of Urology | 2.438 | 0724-4983 |
| 24 | International Journal of Impotence Research | 2.415 | 0955-9930 |
| 25 | International Urogynecology Journal | 2.368 | 0937-3462 |
| 26 | Urology | 2.334 | 0090-4295 |
| 27 | Seminars in Dialysis | 2.269 | 0894-0959 |
| 28 | Prostate Cancer and Prostatic Diseases | 2.263 | 1365-7852 |
| 29 | Pediatric Nephrology | 2.183 | 0931-041X |
| 30 | Urologic Clinics of North America | 2.179 | 0094-0143 |
| 31 | Seminars in Nephrology | 2.154 | 0270-9295 |
| 32 | Bmc Nephrology | 2.136 | 1471-2369 |
| 33 | Advances in Chronic Kidney Disease | 1.843 | 1548-5595 |
| 34 | Nephron Clinical Practice | 1.843 | 1660-2110 |
| 35 | Journal of Endourology | 1.729 | 0892-7790 |
| 36 | Aging Male | 1.674 | 1368-5538 |
| 37 | Clinical Genitourinary Cancer | 1.638 | 1558-7673 |
| 38 | Journal of Nephrology | 1.623 | 1121-8428 |
| 39 | International Urology and Nephrology | 1.567 | 0301-1623 |
| 40 | Asian Journal of Andrology | 1.549 | 1008-682X |
| 41 | Blood Purification | 1.521 | 0253-5068 |
| 42 | Kidney Blood Pressure Research | 1.500 | 1420-4096 |
| 43 | Journal of Renal Nutrition | 1.480 | 1051-2276 |
| 44 | Peritoneal Dialysis International | 1.477 | 0896-8608 |
| 45 | Clinical and Experimental Nephrology | 1.460 | 1342-1751 |
| 46 | International Journal of Urology | 1.460 | 0919-8172 |
| 47 | International Braz J Urol | 1.397 | 1677-5538 |
| 48 | Scandinavian Journal of Urology And Nephrology | 1.293 | 0036-5599 |
| 49 | Contributions to Nephrology | 1.274 | 0302-5144 |
| 50 | Canadian Urological Association Journal | 1.172 | 1911-6470 |
| 51 | Nephrology | 1.172 | 1320-5358 |
| 52 | Urological Research | 1.172 | 0300-5623 |
| 53 | Therapeutic Apheresis and Dialysis | 1.098 | 1744-9979 |
| 54 | Hemodialysis International | 1.093 | 1492-7535 |
| 55 | Clinical Nephrology | 1.058 | 0301-0430 |
| 56 | Urologia Internationalis | 0.924 | 0042-1138 |
| 57 | Canadian Journal of Urology | 0.822 | 1195-9479 |
| 58 | Renal Failure | 0.790 | 0886-022X |
| 59 | Nefrologia | 0.738 | 0211-6995 |
| 60 | Progres En Urologie | 0.481 | 1166-7087 |
| 61 | Urologe | 0.442 | 0340-2592 |
| 62 | Aktuelle Urologie | 0.303 | 0001-7868 |
| 63 | Actas Urologicas Espanolas | 0.274 | 0210-4806 |
| 64 | Dialysis Transplantation | 0.132 | 0090-2934 |
